# Increased levels of the long noncoding RNA, HOXA-AS3, promote proliferation of A549 cells

**DOI:** 10.1038/s41419-018-0725-4

**Published:** 2018-06-13

**Authors:** Hongyue Zhang, Ying Liu, Lixin Yan, Min Zhang, Xiufeng Yu, Wei Du, Siqi Wang, Qiaozhi Li, He Chen, Yafeng Zhang, Hanliang Sun, Zhidong Tang, Daling Zhu

**Affiliations:** 1 College of Pharmacy, Harbin Medical University, Harbin, Heilongjiang China; 2 Central Laboratory of Harbin Medical University-Daqing, Daqing, Heilongjiang China; 3 College of Medical Laboratory Science and Technology, Harbin Medical University-Daqing, Daqing, Heilongjiang China; 4 College of Pharmacy, Harbin University of Commerce, Harbin, Heilongjiang China; 5 Department of Obstetrics and gynecology, The Second affiliated Hospital of Harbin Medical University, Harbin, Heilongjiang China; 6 School of Basic Medical Sciences, Harbin Medical University-Daqing, Daqing, Heilongjiang China; 7 Medical Laboratory Technology, Harbin Medical University-Daqing, Daqing, Heilongjiang China; 8 Department of Medical Informatics, Harbin Medical University-Daqing, Daqing, Heilongjiang China

## Abstract

Many long noncoding RNAs (lncRNAs) have been identified as powerful regulators of lung adenocarcinoma (LAD). However, the role of HOXA-AS3, a novel lncRNA, in LAD is largely unknown. In this study, we showed that HOXA-AS3 was significantly upregulated in LAD tissues and A549 cells. After knockdown of HOXA-AS3, cell proliferation, migration, and invasion were inhibited. Xenografts derived from A549 cells transfected with shRNA/HOXA-AS3 had significantly lower tumor weights and smaller tumor volumes. We also demonstrated that HOXA-AS3 increased HOXA6 mRNA stability by forming an RNA duplex. In addition, HOXA6 promoted cell proliferation, migration, and invasion in vitro. Using a RNA pull-down assay, we found that HOXA-AS3 bonded with NF110, which regulated the cell localization of HOXA-AS3. Moreover, histone acetylation was involved in upregulation of HOXA-AS3. These results demonstrate that HOXA-AS3 was activated in LAD and supported cancer cell progression. Therefore, inhibition of HOXA-AS3 could be an effective targeted therapy for patients with LAD.

## Introduction

Lung cancer is the most common cancer in the world and is associated with high morbidity and mortality^1,2^. Non-small cell lung cancer (NSCLC) now accounts for 70–80% of all lung cancer cases and it is the most common type of lung cancer^3^. Lung adenocarcinoma (LAD), a histological subtype of NSCLC, is now the most common histological type among all diagnosed lung cancers^4^. Despite progress in therapies and advances in its early detection, the prognosis of lung cancer is still not optimistic; the 5-year survival is ~ 16.6%^5,6^ whereas that of NSCLC is 1%^7^ and regional or distant metastasis is the leading cause of poor survival^8–11^. A549 cell proliferation plays an important role in LAD metastasis^12,13^. Although cell proliferation is an important pathophysiological process in the pathogenesis of LAD, its molecular basis remains poorly understood.

The mammalian genome encodes a large number of long noncoding RNAs (lncRNA), which transcribe over 200 nucleotides that have no evidence of protein-coding potential, but increasing evidence suggests that lncRNAs have important biochemical functions^14–17^. Approximately 50–70% of lncRNAs are classified as antisense transcripts (ASTs), defined as RNAs that are reverse complements of their endogenous sense counterparts that frequently do not encode proteins^18–20^. ASTs play a major role in the regulation of its adjacent coding genes. Their effect on other genes include suppression, activation, and homeostatic adjustment, including transcriptional regulation and post-transcriptional regulation^21–24^. ASTs, such as HNF1A-AS1 and SOX21-AS1, have been shown to play a role in the proliferation of LAD cells, but the mechanisms underlying their regulation of adjacent genes have not been established^25,26^.

In our study, we focused on the regulatory effects of one particular AST, HOXA-AS3, in LAD. HOXA-AS3 belongs to the clusters of HOX genes, a group of highly homologous transcription factors that regulate embryological development^27^, and also regulate hematopoietic lineage and differentiation^28^. As a novel lncRNA, there has been only two published studies describing the function of HOXA-AS3: Zhu et al.^29^ reported that HOXA-AS3 interacted with EZH2 to regulate lineage commitment of mesenchymal stem cells. And in glioma, upregulation of HOXA-AS3 promotes tumor progression and predicts poor prognosis. Other members of the HOXA cluster, such as HOTAIR and HOTTIP, have been reported to play a role in regulating cell proliferation in lung cancer^30^. But there is no report on the role of HOXA-AS3 in lung cancer. In addition, there is limited knowledge regarding the mechanism by which HOXA-AS3 functions as an AST.

Our study identified a critical function of HOXA-AS3 in LAD and provided new evidence for an improved understanding of the role of lncRNAs in A549 cells proliferation.

## Materials and methods

For detailed Material and Methods, please see the Supplementary Data 1.

### Statistical analysis

The composite data were expressed as mean ± SEM. Statistical analysis was performed with ANOVA followed by Dunnett’s test or Student’s *t* test or Pearson correlation test. Differences were considered to be significant at *P* ≤ 0.05.

## Results

### HOXA-AS3 is upregulated in LAD and A549 cells

NCBI database has validated two HOXA-AS3 transcripts, transcript variant 1 (TV-1) and TV-2. To investigate the role of each individual transcript in LAD, specific primers were designed to target the various transcript variants. quantitative polymerase chain reaction (qRT-PCR) showed that expressions of TV-1 and TV-2 were elevated significantly as compared with that in adjacent tissues and TV-1 increased more significantly (Supplementary Data 2, Figure S1A). In addition, the expression of TV-1 in A549 cells also showed notable upregulation, compared with the expression of TV-2 (Figure S1B). Thus, we will focus on TV-1 for our study, and it will be referred as HOXA-AS3 thereafter. We transiently transfected the Flag-tagged expression vectors into A549 cells and immunoblotting showed that Flag is hardly detected in HOXA-AS3 group. However, it is easily detectable in GAPDH group (Figure S1C). Bioinformatics analysis also confirmed HOXA-AS3 had no coding capability (http://cpc.cbi.pku.edu.cn/programs/run_cpc.jsp). So we conclude that HOXA-AS3 is a lncRNA.

**Fig. 1:**
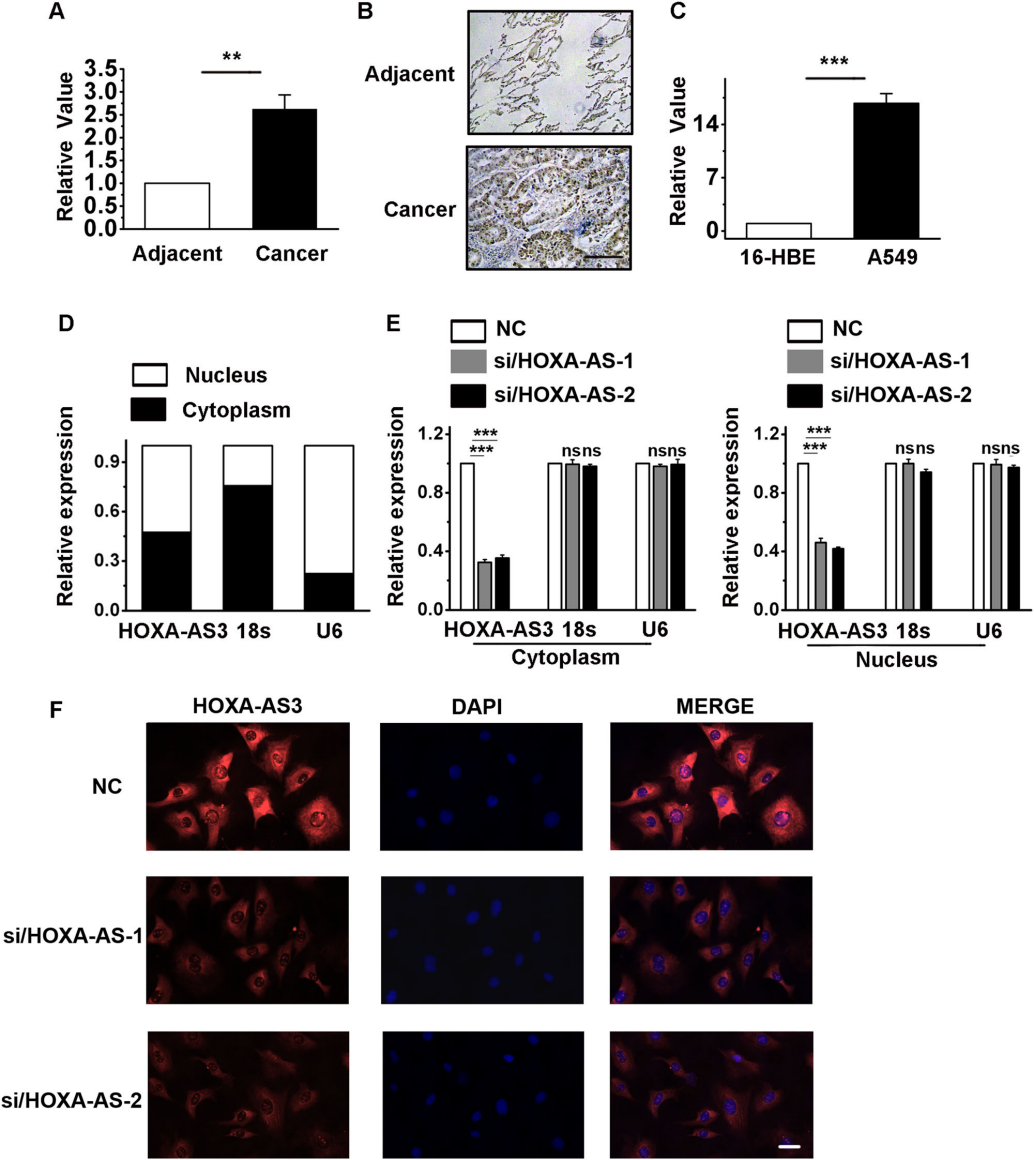
The expression of HOXA-AS3 in lung adenocarcinoma (LAD) and A549 cells. **a** Expression of HOXA-AS3 quantified by the quantitative polymerase chain reaction (qRT-PCR) in LAD samples and their corresponding adjacent normal tissues. **b** In situ hybridization of HOXA-AS3 RNA expression in tumor tissue samples of LAD. Scale bar, 50 μm. **c** Expression of HOXA-AS3 quantified by qRT-PCR in human LAD cell line, A549, and in a normal bronchial epithelial cell line, 16HBE. **d** Representative analysis of the HOXA-AS3 distribution by cellular fractionation in A549 cells. U6 mRNA and 18 s mRNA were controls for nuclear and cytoplasmic RNAs, respectively. **e** Expression of HOXA-AS3 quantified by the qRT-PCR in the nuclear and cytoplasmic fractions from NC and siRNA/HOXA-AS3 cells. **f** RNA-fluorescent in situ hybridization (FISH) was performed to detect HOXA-AS3 expression in A549 cells. The nuclei were counterstained with 4′,6-diamidino-2-phenylindole (DAPI). Scale bar, 50 μm. Adjacent, LAD-corresponding to adjacent normal tissue; cancer, LAD tissue; ^*^P < 0.05, ^**^P < 0.01, and ^***^P < 0.001. All of the values are expressed as the mean ± SEM

We assessed HOXA-AS3 expression in LAD samples and their corresponding adjacent normal tissues. In cancerous tissues, HOXA-AS3 expression was higher than the average level observed in normal tissues (Fig. 1a). In total, strong HOXA-AS3 transcript expression was observed in LAD tissues, whereas in nonmalignant normal lung tissue, the HOXA-AS3 transcript was barely detectable (Fig. 1b). We determined the RNA expression of HOXA-AS3 in the human LAD cell line, A549, and the normal bronchial epithelial cell line, 16HBE. There was significantly increased HOXA-AS3 RNA in A549 cells (Fig. 1c). We next performed cellular fractionation to analyze the subcellular localization of HOXA-AS3 in A549 cells. HOXA-AS3 was almost evenly distributed in the cytoplasm and nucleus (Fig. 1d). To further investigate the function of increased HOXA-AS3 levels in A549 cells, small interfering RNA (siRNA) was targeted to HOXA-AS3. As expected, transduction with siRNA/HOXA-AS3 resulted in low human HOXA-AS3 expression in A549 cells (Figure S2A). Figure 1e showed that siRNA has successfully knockdown the expression of HOXA-AS3 in nuclear and cytoplasmic fractions. The results were further confirmed by RNA-fluorescent in situ hybridization, showing that HOXA-AS3 expression was distributed in both the cytoplasm and nucleus of A549 cells (Fig. 1f).

### HOXA-AS3 promotes cell proliferation in A549 cells

**Fig. 2:**
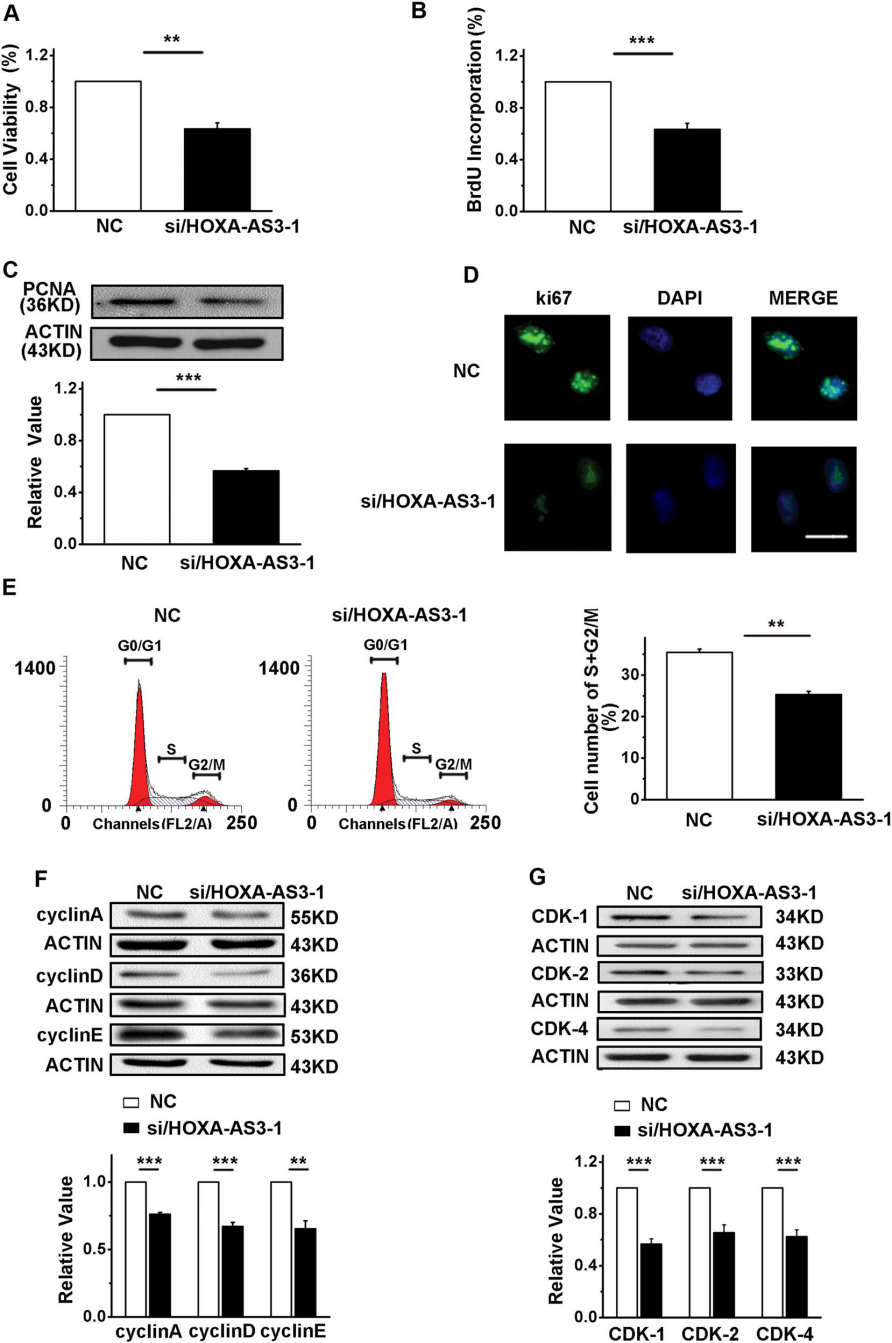
HOXA-AS3 knockdown reduces cell proliferation and regulates the cell cycle. **a** The 3-(4,5-dimethylthiazol-2-yl)-2,5-diphenyltetrazolium bromide (MTT) assay was used to determine the cell viability in A549 cells. **b** 5-bromodeoxyuridine incorporation showed the synthesis of DNA. **c** Western blot analyses of the protein expression levels of proliferating cell nuclear antigen. **d** Immunofluorescence for ki67 in A549 cells, after staining the nuclei with 4′,6-diamidino-2-phenylindole (DAPI). Scale bar, 25 μm. **e** Left, fluorescence-activated cell sorting (FACS) analyses detected the number of cells in each phase of the cell cycle. Right, the percentage of cells in the S and G2/M phases in A549 cells. **f** Western blot analyses of the protein expression levels of cyclin (**a**), cyclin (**d**), and cyclin (**e**). **g** Western blot analyses of the protein expression levels of CDK1, 2, and 4. NC, negative control; siRNA/HOXA-AS3, small interfering RNA for HOXA-AS3. ^*^P < 0.05, ^**^P < 0.01, and ^***^P < 0.001. All values are expressed as the mean ± SEM

When HOXA-AS3 was silenced, cell viability (Fig. 2a) and proliferation (Fig. 2b) in A549 cells decreased. In addition, we detected the expression of the proliferating cell nuclear antigen (PCNA) in A549 cells. After HOXA-AS3 was knocked down, the expression of PCNA was significantly decreased (Fig. 2c). A similar phenotype was observed in the expression of ki67 (Fig. 2d).

### HOXA-AS3 regulates cell proliferation by accelerating the cell cycle

To investigate the effects of HOXA-AS3 on the cell cycle, the number of cells in different cell cycle stages was detected by flow cytometry. As shown in Fig. 2e, the percentage of cells in S + G2/M phases was decreased in A549 cells in which HOXA-AS3 was knocked down. After siRNA transfection for HOXA-AS3 in A549 cells, the expression levels of cyclin A, cyclin D, and cyclin E were decreased (Fig. 2f). We also examined the expression of cyclin-dependent protein kinases 1, 2, and 4 in A549 cells, which were downregulated after knocking down the HOXA-AS3 gene (Fig. 2g). We used another independent siRNA to knockdown HOXA-AS3, and obtained the same results as above (Figure S2B–H).

### HOXA-AS3 promotes cell migration, invasion, and colony formation in vitro

**Fig. 3:**
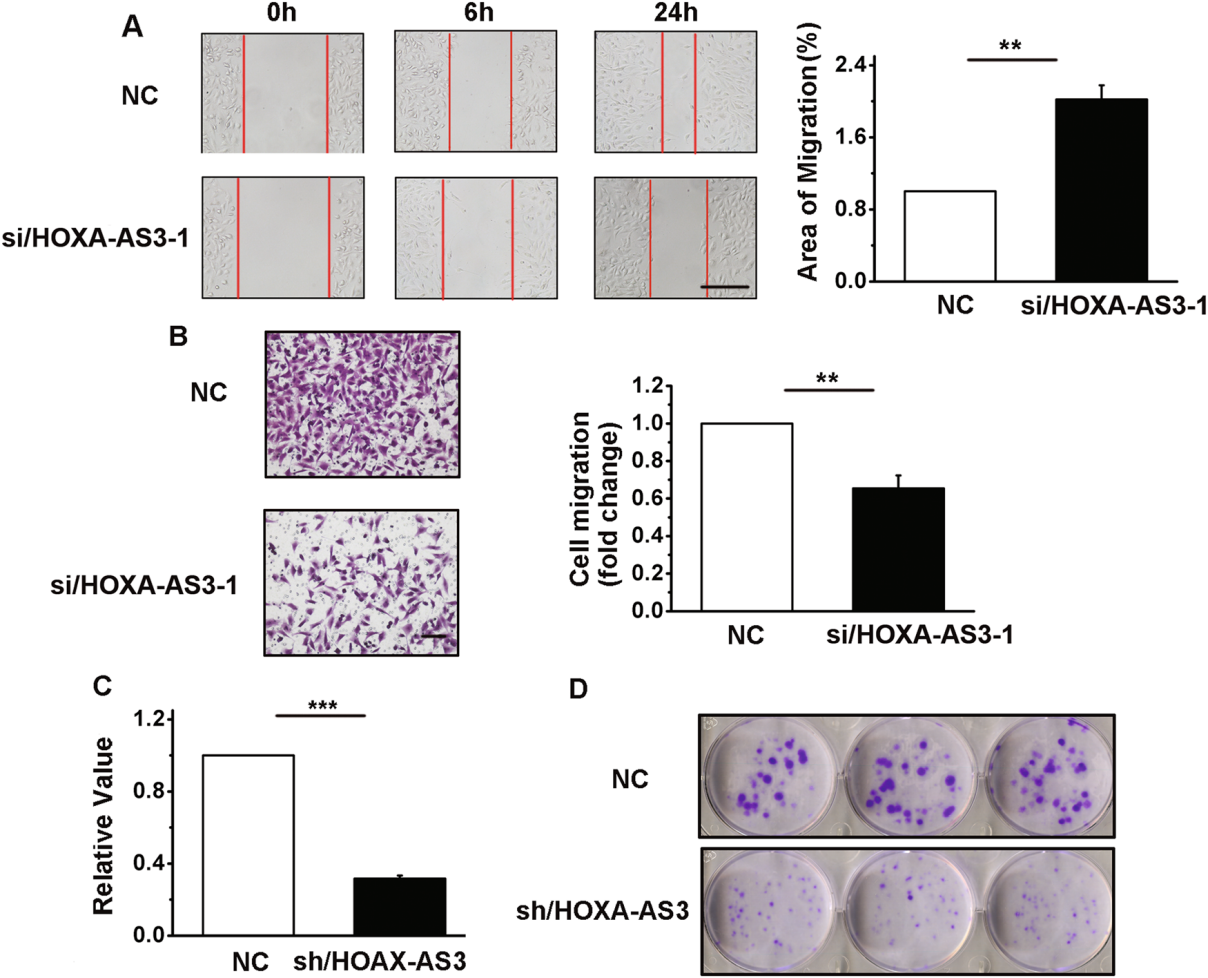
HOXA-AS3 knockdown inhibits cell migration, invasion, and colony formation in A549 cells. **a** Left, A549 cells were subjected to a scratch-wound assay. Scale bar, 50 μm. Right, the histogram shows the cell migration ability of A549 cells. **b** Left, A549 cells were subjected to Matrigel^®^ invasion chamber analyses. Scale bar, 50 μm. Right, the histogram shows the cell invasion ability of A549 cells. **c** Determination of the efficiency of shRNA directed against HOXA-AS3 using the quantitative polymerase chain reaction (qRT-PCR). **d** A colony forming growth assay was performed to determine the proliferation in A549 cells. NC, negative control; siRNA/HOXA-AS3, small interfering RNA for HOXA-AS3; shRNA/NC, small hairpin RNA for negative control; shRNA/HOXA-AS3, small hairpin RNA for HOXA-AS3. ^*^P < 0.05, ^**^P < 0.01, and ^***^P < 0.001. All values are expressed as the mean ± SEM

The migration and invasive capacities of A549 cells were markedly decreased when HOXA-AS3 was blocked by siRNA (Fig. 3a, b and Figure S3A-B). To further investigate the role of HOXA-AS3 in LAD tumorigenesis, we established a stably HOXA-AS3 downregulated cell line, by using small hairpin RNA (shRNA)-HOXA-AS3, and shRNA-NC-transfected (control) A549 cells. As shown in Fig. 3c, there was a reduction of approximately 70% in HOXA-AS3 expression in the shRNA-HOXA-AS3-transfected A549 cells compared with that in the shRNA-NC-transfected cells. Next, Fig. 3d shows that colony formation ability was decreased following HOXA-AS3 knockdown in A549 cells.

### HOXA-AS3 promotes tumor growth in vivo

**Fig. 4:**
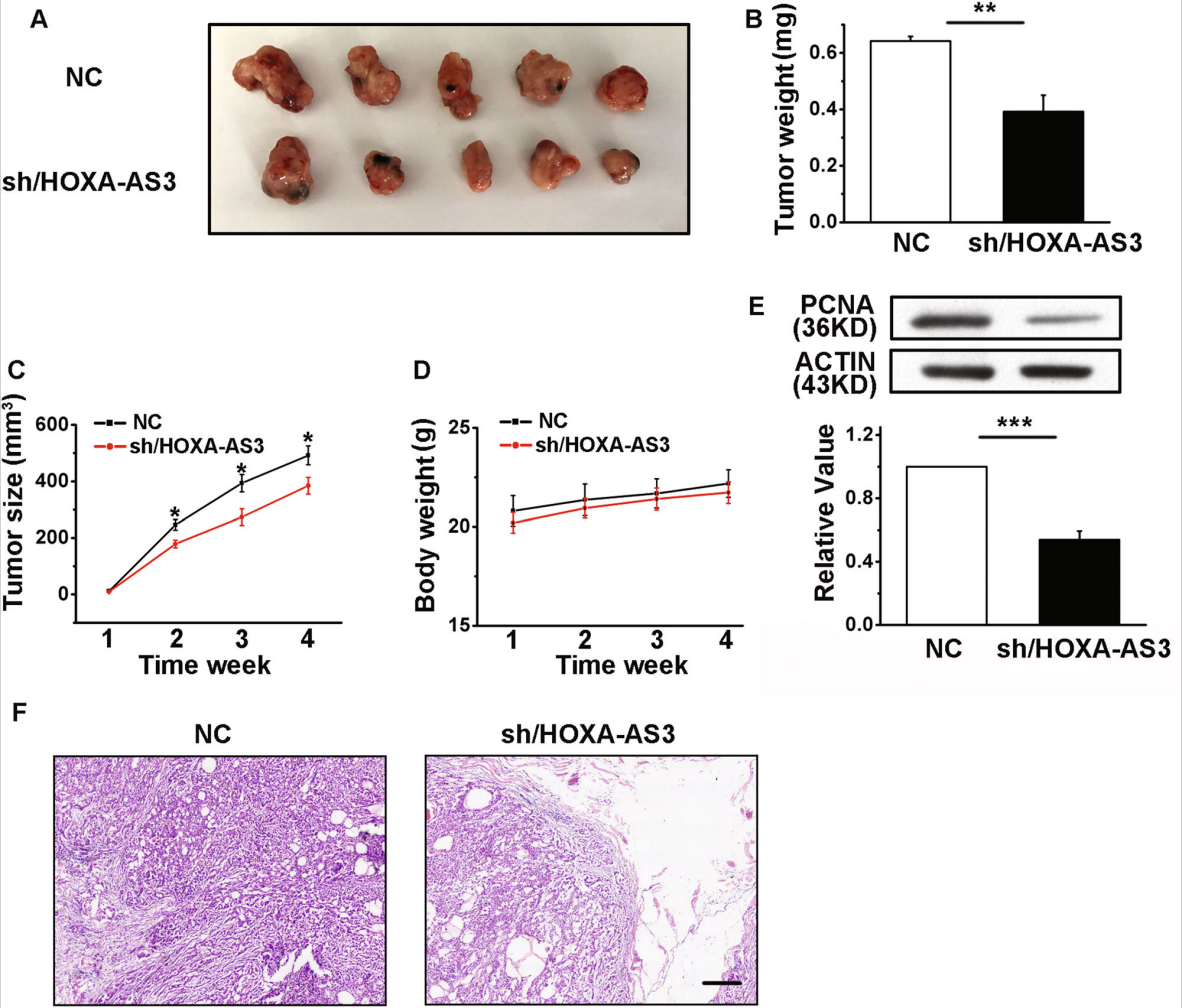
HOXA-AS3 knockdown inhibits xenograft tumor growth in nude mice. **a** Photograph of xenograft tumors derived from nude mice. **b** Tumor weight when the tumors were harvested. **c** Tumor growth curve. Five mice per group were used in the xenograft experiment, and the tumor volumes of mice were measured. **d** The body weight of the nude mice. **e** Western blot analyses of the protein expression levels of proliferating cell nuclear antigen. **f** Hematoxylin and eosin staining for tumor tissues from mice. Scale bar, 50 μm. shRNA/NC, small hairpin RNA for the negative control; shRNA/HOXA-AS3, small hairpin RNA for HOXA-AS3. ^*^P < 0.05, ^**^P < 0.01, and ^***^P < 0.001. All values are expressed as the mean ± SEM

A549 cells transfected with either shRNA/NC or shRNA/HOXA-AS3 were subcutaneously injected into nude mice. Tumors derived from shRNA-HOXA-AS3 had significantly lower tumor weights and smaller tumor volumes compared with shRNA/NC mice (Fig. 4a–c). Moreover, we were concerned about the health and body weight of the mice, but found that there was no obvious toxicity after interfering with HOXA-AS3 (Fig. 4d). The expression of PCNA in shRNA/HOXA-AS3 mice tissue was significantly decreased compared with that of the shRNA/NC mice (Fig. 4e). We then performed histological examinations using hematoxylin and eosin staining to determine the degree of LAD differentiation. The results showed that shRNA/HOXA-AS3-treated mice exhibited well differentiated adenocarcinomas (Fig. 4f).

### HOXA-AS3 increases HOXA6 mRNA stability by forming an RNA duplex

**Fig. 5:**
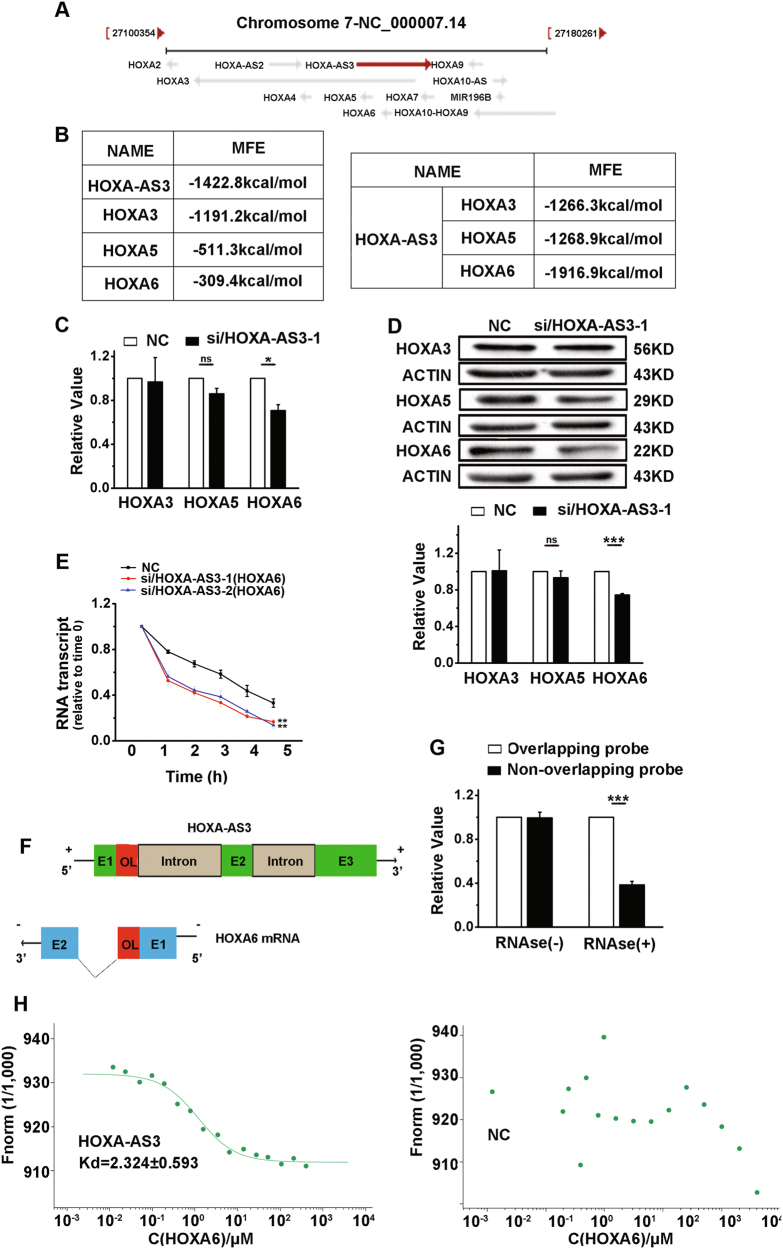
HOXA-AS3 and HOXA6 mRNAs form a duplex RNA-RNA structure at their mutually overlapping regions. **a** Genomic sequences of HOXA-AS3. Arrows show the direction of the transcription. **b** RNAfold and RNAcofold predict the binding capacity and the binding free energy, respectively, of HOXA-AS3 with its neighboring genes. **c** Expression of HOXA3, HOXA5, and HOXA6 quantified by qRT-PCR after HOXA-AS3 knockdown in A549 cells. **d** Western blot analyses of the protein expression levels of HOXA3, HOXA5, and HOXA6 after HOXA-AS3 knockdown in A549 cells. **e** The stability of HOXA6 mRNA over time was measured by qRT-PCR relative to time 0 after 5 μg/ml actinomycin (**d**) treatment in A549 cells transiently expressing HOXA-AS3 siRNAs or control siRNA. **f** The start and end positions of genes as indicated on the UCSC site (Genome Bioinformatics). The black arrows indicate the transcription direction. The schema in scale “+” at both sides of the strand represent the positive strand, and “−” represent the negative strand. Blocks with colors (green, blue, and red) represent exons. The red block in the HOXA-AS3 transcript represents the overlapping region (OL). The red block in HOXA6 mRNA represents the first exon and the OL region. “**e**” followed by a number denotes the exon and its serial number. The gray block represents the intronic portion of HOXA-AS3. The dotted filled lines between colored blocks represent introns in HOXA6. **g** Recombinase polymerase amplification (RPA) performed on RNA samples from A549 cells. Depicted are qRT-PCR results from two sets of primers and probes covering overlapping and nonoverlapping regions of HOXA6 mRNA. **h** Determination of the binding affinity of HOXA6 to HOXA-AS3 mRNA by microscale thermophoresis. The concentration of labeled HOXA-AS3 molecules was constant, whereas the concentrations of the nonlabeled binding partner HOXA6 molecules varied from 10 nM to 400 μM. NC, negative control; siRNA/HOXA-AS3, small interfering RNA for HOXA-AS3. ^*^P < 0.05, ^**^P < 0.01, and ^***^P < 0.001. All values are expressed as the mean ± SEM

Using the UCSC genome browser, HOXA-AS3, an lncRNA, was located between and antisense to the human *HOXA3, HOXA5, HOXA6* genes (Fig. 5a). One of the mechanisms for AST-mediated gene regulation was based on AS RNAs forming duplexes with their neighboring mRNAs, which protected them from ribonuclease degradation^31,32^. We applied the RNA thermodynamic structure prediction (http://rna.tbi.univie.ac.at/), which predicts the binding capacity and the binding free energy of RNAs. We found that antisense lncRNA-HOXA-AS3 with HOXA6 formed double-stranded RNA, which exhibited lower levels of minimum free energy, suggesting that the double-stranded structure was more stable (Fig. 5b). In addition, the HOXA6 mRNA and protein levels were decreased after HOXA-AS3 knockdown in A549 cells (Fig. 5c, d and Figure S4A–B), and the expression of HOXA3 and HOXA5 mRNA and protein were unchanged. We assessed the stability of HOXA3, HOXA5, and HOXA6 transcripts by quantifying the levels of mRNA that remained in the presence of actinomycin D. We found decreased stability of HOXA6 mRNA in siRNA/HOXA-AS3 cells compared with cells transfected with a control siRNA (Fig. 5e). And the stability of HOXA3 and HOXA5 transcripts were unchanged (Figure S4C).

Figure 5f and Figure S4D show that HOXA-AS3 shares its first exon with the first exon of HOXA6 (from the UCSC site), which we refer to as the overlapping region (OL). We then used an RNase protection assay to verify that HOXA-AS3 and HOXA6 mRNAs formed a protective duplex, specifically at their OLs. Total RNA from A549 cells was used to test this hypothesis. The PCR results showed that the OL was partially protected from RNase degradation, indicating that HOXA6 and HOXA-AS3 formed an RNA duplex (Fig. 5g and Figure S4E). Recombinase polymerase amplification (RPA) and RT-PCR were also performed on the nuclear RNA from A549 cells and results showed that the OL was not protected from RNase degradation, thus eliminating the possibility that RNA duplex forms in the nucleus (Figure S4F). The interaction of the fluorescently labeled HOXA-AS3 or the negative control (NC) probe molecules with the unlabeled competitor molecule, HOXA6, was assayed using microscale thermophoresis (MST), which facilitated sensitive measurements of molecular interactions in solution^33,34^. The results showed that HOXA-AS3 bound to HOXA6 at low micromolar concentrations of the titrant, exhibiting a dissociation constant (*K*_d_) of 2.323 ± 0.593 μM (Fig. 5h, left), suggesting a relatively strong interaction. In contrast, the NC displayed a complete defect in binding to HOXA6 mRNA (Fig. 5h, right) indicating that HOXA6 mRNA was a direct target of the HOXA-AS3.

### HOXA6 promotes cell proliferation, migration, and invasion in vitro

**Fig. 6:**
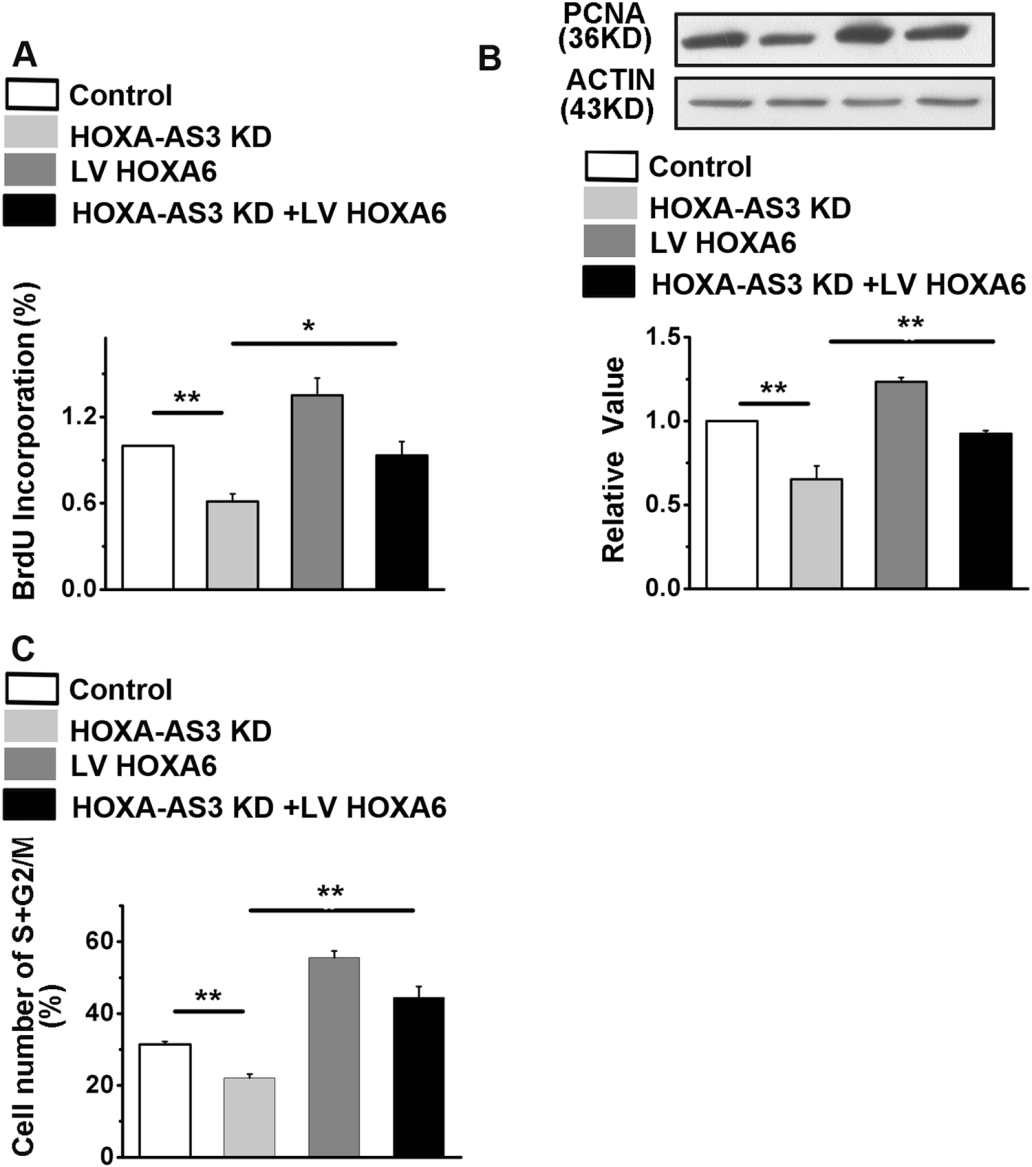
Rescue effect on proliferation and cell cycle regulation by HOXA6 overexpression in HOXA-AS3 knock down cells. **a** 5-bromodeoxyuridine incorporation showed the synthesis of DNA. **b** Western blot analyses of the protein expression levels of proliferating cell nuclear antigen. **c** Fluorescence-activated cell sorting (FACS) analyses to determine the percentage of cells in S and G2/M phases in A549 cells. ^*^P < 0.05, ^**^P < 0.01, and ^***^P < 0.001. All values are expressed as the mean ± SEM

We detected the expression of HOXA6 mRNA in LAD tissues and their corresponding adjacent normal tissues. In cancerous tissues, HOXA6 expression was higher than the average level observed in normal tissues (Figure S5A). Notably, in the same samples, HOXA6 mRNA levels were much higher than HOXA-AS3 levels. We found that levels of HOXA-AS3 and HOXA6 mRNA were positively correlated in LAD tissues (*R* = 0.633, *P* < 0.05) (Figure S5B). In addition, HOXA6 knockdown did not affect the expression of HOXA-AS3 (data not shown). We suspected that HOXA-AS3 might promote cancer cell proliferation migration and invasion by interacting with HOXA6, so we further characterized the function of HOXA6 in A549 cells, and the efficiency of HOXA6 silencing was determined (Figure S5C). As shown in Figure S5D, the percentage of cells in S + G2/M phases was decreased in A549 cells in which HOXA6 was knocked down. Furthermore, after siRNA transfection for HOXA6 in A549 cells, the expression levels of cyclin A, cyclin D, and cyclin E decreased, and the expression of PCNA decreased after HOXA6 knockdown. We also detected the expression of the tumor suppressor gene, p53, after HOXA6 knockdown. Figure S5E shows that the expression of P53 was upregulated. Furthermore, the invasive capacity of the A549 cells was significantly decreased when HOXA6 was blocked by siRNA (Figure S5F). To further define the relationship between HOXA-AS3 and HOXA6, we attempted to rescue the decreased proliferation induced by HOXA-AS3 knockdown by overexpressing HOXA6. To this end, we infected HOXA-AS3-knockdown A549 cells with viruses expressing HOXA6. The stable cell lines with HOXA6 expression higher than normal expression levels were selected for further analysis (Figures S5G). We found that overexpression of HOXA6 in A549 cells partially restores decreased proliferation induced by HOXA-AS3 knockdown (Fig. 6a). Similar results are found in the expression of PCNA (Fig. 6b). Fluorescence-activated cell sorting analysis demonstrated that the decreased percentage of cells in S + G2/M phases caused by HOXA-AS3 knockdown is successfully rescued by expression of HOXA6 (Fig. 6c).

### NF110 regulates HOXA-AS3 subcellular distribution in A549 cells

**Fig. 7:**
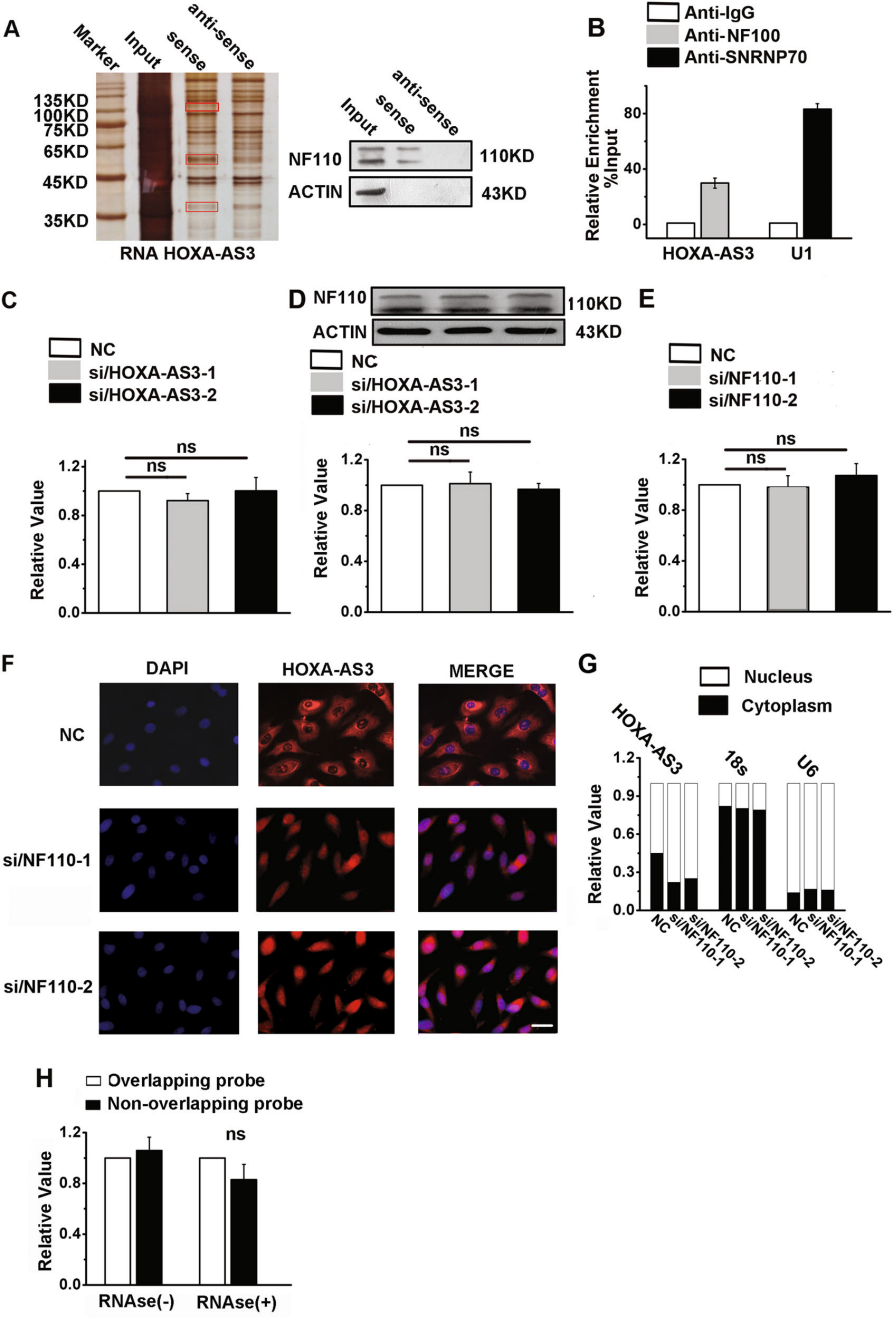
HOXA-AS3 interacts with NF110. **a** Left, identification of HOXA-AS3-associated target proteins in A549 cells using a RNA pull-down assay. The bands specific to sense HOXA-AS3 were detected using mass spectrometry. Right, western blot analyses of the interaction of HOXA-AS3 with NF110. **b** RNA immunoprecipitation experiments were performed using NF110 antibody, SNRNP70 antibody and IgG antibody in A549 cells. qRT-PCR was performed to detect pulled-down HOXA-AS3 and U1. SNRNP70 antibody and IgG antibody were used as positive and negative controls, respectively. **c** Expression of NF110 quantified by qRT-PCR after HOXA-AS3 knockdown in A549 cells. **d** Western blot analyses of the protein expression levels of NF110 after HOXA-AS3 knockdown in A549 cells. **e** Expression of HOXA-AS3 quantified by qRT-PCR after NF110 knockdown in A549 cells. **f** RNA-FISH was performed to detect HOXA-AS3 expression in A549 cells. Nuclei were counterstained with DAPI. Scale bar, 25 μM. **g** Representative analysis of HOXA-AS3 distribution by cellular fractionation of A549 cells. U6 mRNA and 18 s mRNA were the controls for nuclear and cytoplasmic RNAs, respectively. **h** RPA performed on RNA samples from A549 cells that knocked down NF110. Depicted are qRT-PCR results from two sets of primers and probes covering overlapping and nonoverlapping regions of HOXA6 mRNA. NC, negative control; siRNA/HOXA-AS3, small interfering RNA for HOXA-AS3; siRNA/NF110, small interfering RNA for NF110. ^*^P < 0.05, ^**^P < 0.01, and ^***^P < 0.001. All values are expressed as the mean ± SEM

We then identified proteins that were associated with HOXA-AS3 using an RNA pull-down assay, as previously described^35^. The bands specific to lncRNA-HOX-AS3 were excised and subjected to mass spectrometry (Fig. 7a, left). Among the proteins identified by mass spectrometry, only NF110 was detected by western blotting from three independent RNA pull-down assays (Fig. 7a, right), suggesting a biochemical/physiological function for the association between HOXA-AS3 and NF100. immunoprecipitation(RIP) results also showed that HOXA-AS3 interacts with NF110 protein in A549 cells (Fig. 7b). Moreover, the mRNA and protein expression of NF110 changed little upon HOXA-AS3 knockdown in A549 cells (Fig. 7c, d), and NF110 knockdown did not affect the expression of HOXA-AS3 (Fig. 7e) and TV-2 (Figure S6B). Because NF110 has been implicated in the stabilization, transport, and translational control of many target mRNAs^36^, the possible effect of NF110 on HOXA-AS3 subcellular distribution was characterized by determining the ratio of cytoplasmic and nuclear HOXA-AS3. In A549 cells, HOXA-AS3 evenly distributed in the cytoplasm and nucleus; however, after knockdown of NF110 in A549 cells, HOXA-AS3 diffusely distributed throughout the nucleus (Fig. 7f, g), suggesting that NF110 regulated HOXA-AS3 subcellular distribution in A549 cells. In addition, NF110 knockdown decreased the expression of HOXA6 in A549 cells (Figure S6C). We also extracted RNA from A549 cells that knocked down NF110 and performed RPA (Fig. 7h). The results showed that under the action of RNase, protective RNA duplex formed by HOXA-AS3 and HOXA6 hardly existed in NF110 knockdown A549 cells.

### Upregulation of HOXA-AS3 by histone acetylation in A549 cells

**Fig. 8:**
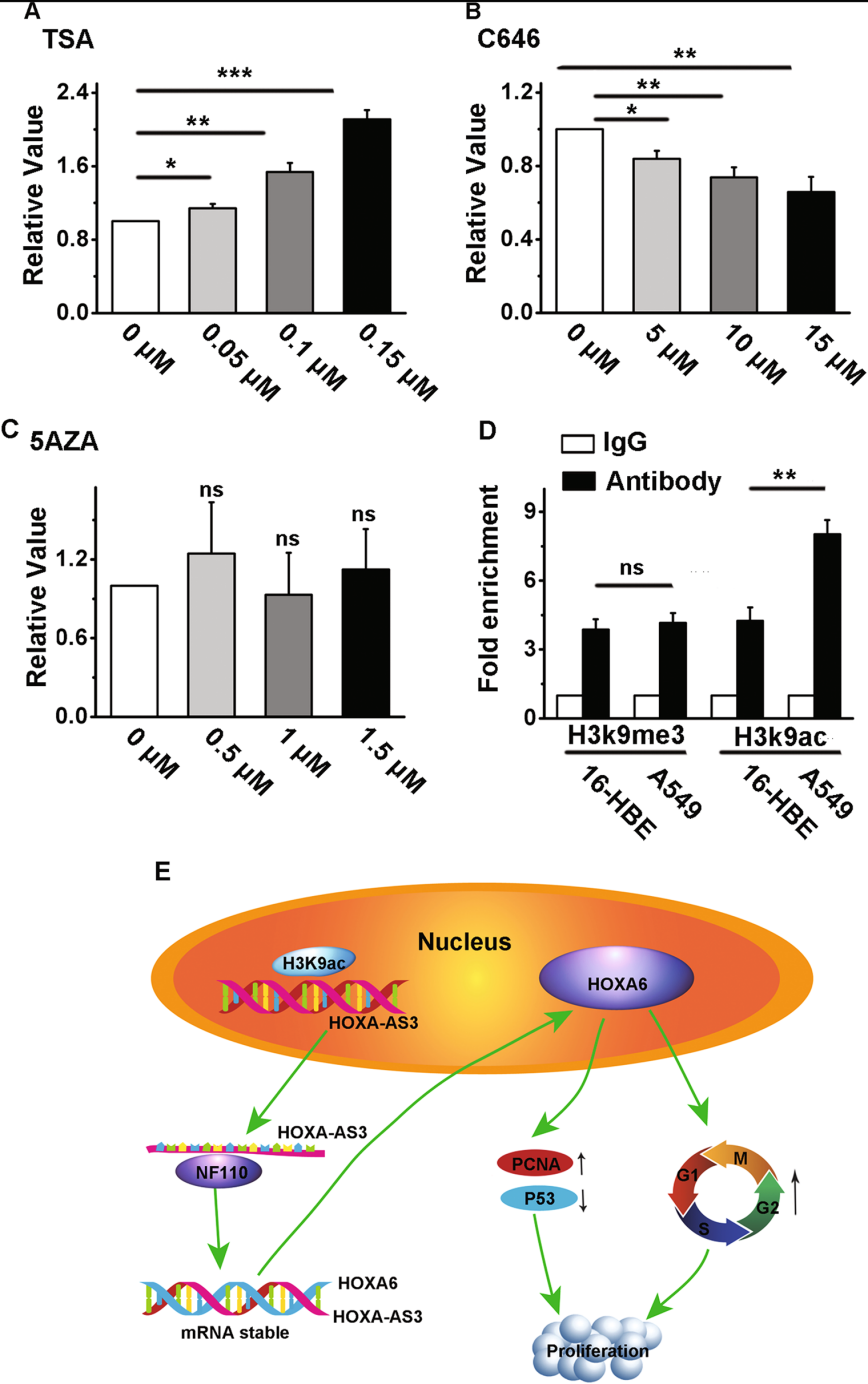
Upregulation of HOXA-AS3 by histone acetylation in A549 cells. **a** A549 cells were stimulated with varying concentrations of trichostatin A (TSA) for 24 h. **b** A549 cells were stimulated with varying concentrations of C646 for 24 h. **c** A549 cells were stimulated with varying concentrations of 5-aza-2′-deoxycytidine (5-AZA) for 24 h. **d** Binding of histone modifications to the HOXA-AS3 promoter region was examined using a chromatin immunoprecipitation (ChIP) assay in A549 cells. **e** Summary diagram describing how HOXA-AS3 regulates proliferation in A549 cells. ^*^P < 0.05, ^**^P < 0.01, and ^***^P < 0.001. All values are expressed as the mean ± SEM

Epigenetic modifications, especially histone methylation or acetylation, play important roles in tissue-specific gene expression, so lncRNAs might be regulated in a similar manner as protein-coding genes^37^. Epigenetic modifications of HOXA-AS3 should therefore be investigated to identify the mechanism of upregulation of HOXA-AS3 in A549 cells. HOXA-AS3 was upregulated by the histone deacetylase inhibitor, trichostatin A in A549 cells (Fig. 8a). Notably, the expression of HOXA-AS3 was downregulated by histone acetyltransferase p300 inhibitor, C646 (Fig. 8b). However, 5-aza-2′-deoxycytidine, a cytidine analog and methyltransferase inhibitor, did not alter the expression of HOXA-AS3 in A549 cells (Fig. 8c), suggesting that only histone acetylation was involved in upregulation of HOXA-AS3. A chromatin immunoprecipitation assay was then performed to examine the interaction between the histones and HOXA-AS3. The results showed that the binding of the methylation-related histone, H3K9me3, to the HOXA-AS3 promoter region was similar in both A549 and normal cells, while binding of the acetylation-related histone, H3K9ac, increased significantly (Fig. 8d). We also examined the interaction between the histone modification and HOXA6. Results showed that HOXA-AS3 knockdown did not affect the histone modification of HOXA6 promoter region (Figure S7A-C).

## Discussion

Recent findings have confirmed that many lncRNAs have important roles in lung cancers. Two well-known lncRNAs, MALAT1 and HOTAIR, have been shown to regulate tumorigenesis and metastasis of NSCLC in many reports^38–40^. In addition, increasingly more lncRNAs have been found to regulate the proliferation of A549 cells^41–43^. In the present study, we discovered and assessed a novel LAD-associated lncRNA, namely HOXA-AS3. Our results may provide the missing piece of the well-known oncogenic and tumor suppressor network puzzle.

Obtained results indicated that HOXA-AS3 was significantly upregulated in LAD tissues and A549 cells. HOXA-AS3 participates in cell proliferation and cell cycle regulation. Besides, we revealed that knockdown of HOXA-AS3 inhibits tumorigenesis and cancer progression. Next, we explored the biological mechanisms underlying this inhibition. Considering that HOXA-AS3 is an AST, and that previous studies have reported that natural antisense transcripts have important roles in many physiological or pathological processes via the regulation of sense gene expressions^31,44,45^, we detected the expression of its adjacent coding genes HOXA3, HOXA5, and HOXA6 after HOXA-AS3 knockdown. In addition, as indicated by the bioinformatics analysis, as well as by the results of the RNase protection assay and MST, we confirmed that HOXA-AS3, a natural HOXA6 antisense transcript, upregulated HOXA6 expression at both the mRNA and protein levels, by increasing the stability of HOXA6 mRNA.

Recent reports have highlighted the importance of the HOXA cluster in tumorigenesis and leukemogenesis^46–48^. In particular, HOXA6 is one of a subset of HOX genes upregulated in patients with acute myeloid leukemia^49^. Similarly, we found that HOXA6 was significantly upregulated in LAD tissues and promoted cell proliferation, migration, and invasion in vitro. Notably, we found that HOXA6 not only regulated the expression of PCNA, but also that of P53. In addition, we found that the levels of HOXA-AS3 and HOXA6 mRNA were positively correlated in LAD tissues. Consequently, we suspected that the effects of HOXA-AS3 on tumor cell proliferation were mediated by changes in the levels of HOXA6.

Several studies have recently reported that many lncRNAs are involved in molecular regulatory pathways via their interactions with proteins^50,51^. We hypothesized that lncRNA-HOXA-AS3 affects cellular functions in a similar manner. Interleukin enhancing factor 3 (ILF3) is a known transcription factor, which has two main isoforms (NF90 and NF110). They participate in many aspects of RNA metabolism, including transcription, degradation, and translation^52,53^. We found that HOXA-AS3 interacted with NF110, but did not affect its expression. In addition, interfering with NF110 did not impact the expression of HOXA-AS3, but affected its cytoplasmic and nuclear distribution. Gwizdek et al. reported that the second double-stranded RNA-binding domain of ILF3-mediated nuclear export in a complex with adenoviral VA1-RNA, RanGTP, and exportin-5^54^. Our results are consistent with these findings in that NF110 regulated the nucleocytoplasmic shuttling of HOXA-AS3. To the best of our knowledge, this is the first report of the interaction of NF110 with a lncRNA.

In this study, we showed for the first time that histone acylation promotes HOXA-AS3 expression. Epigenetic regulation provides a new perspective for lncRNA regulation. However, available data on this subject are limited, and further studies are needed to reveal the underlying mechanism in detail.

In summary, Fig. 8e depicts the involvement HOXA-AS3 in cell proliferation and cell cycle regulation. H3K9ac binds to the HOXA-AS3 promoter region, where histone acetylation of promoter regions leads to promotion of gene expression. Then, NF110 binds to HOXA-AS3 and regulates its nucleocytoplasmic shuttling. Subsequently, HOXA-AS3 exerts its function by forming an RNA-RNA duplex with its cognate sense transcript, to increase HOXA6 at both the mRNA and protein levels. Finally, the high expression of HOXA6 promotes cell cycle progression and tumorigenesis. To our knowledge, this is the first report of the involvement of HOXA-AS3 in LAD, and until now there has been no report on the mechanism by which HOXA-AS3 regulates cell proliferation. The activation of its adjacent coding genes may serve as an important mechanism underlying the functions of HOXA-AS3.

## Conclusion

In conclusion, our results showed that HOXA-AS3 plays an important role in the proliferation of A549 cells. Therefore, identifying the underlying mechanism could lead to a potential novel strategy for the treatment of LAD.

## Electronic supplementary material

Supplmentary Data 1

Supplmentary Data 2
