# Supplementary material for: Increased levels of the long noncoding RNA, HOXA-AS3, promote proliferation of A549 cells
Source: Cell Death Dis. 2018 Jun 13;9(6):707. doi: 10.1038/s41419-018-0725-4 (PMC5999602; doi:10.1038/s41419-018-0725-4)
Supplement: Supplementary file 1 — Supplmentary Data 1 [file 41419_2018_725_MOESM1_ESM.docx]

**Materials and Methods**

**Clinical specimens**

We obtained nine paired LAD and adjacent normal lung tissues from patients who underwent surgery at the Fifth Affiliated Hospital of Harbin Medical University, China, who were diagnosed with LAD based on histopathological evaluations. Clinicopathological characteristics, including tumor/node/metastasis (TNM) staging were recorded. No local or systemic treatment was conducted in these patients before surgery. All specimens were obtained under sterile conditions during surgery, snap frozen in liquid nitrogen, and stored at -80°C. Our study was approved by the Research Ethics Committee of Harbin Medical University, China, and written informed consent was obtained from all patients.

**Cell lines and cell cultures**
Human lung adenocarcinoma cell lines, A549 and HBE, were obtained from the Institute of Biochemistry and Cell Biology of the Chinese Academy of Sciences, Shanghai, China. The cells were cultured in 10% fetal bovine serum (FBS)-DMEM at 37 °C in a 5% CO_2_ humidified incubator.

**In situ hybridization**

Digoxigenin-labeled DNA probes complementary to HOXA-AS3 RNA were generated using random primer labeling (Boster, Wuhan, China). For in situ hybridization, 5 μm thick LAD sections were prepared and treated.

**RNA isolation and qRT-PCR analyses**

To investigate the expression of target genes, total RNA was extracted and isolated from tissues and cells using TRIzol reagent (Invitrogen, Carlsbad, CA, USA) according to the manufacturer’s protocol. For the qRT-PCR assay, the isolated RNA was reverse-transcribed into cDNA using a reverse transcription kit (Takara, Dalian, China). The expression levels were normalized against the expression of glyceraldehyde 3-phosphate dehydrogenase (GAPDH). The PCR primers were as follows: HOXA-AS3 (TV-1) forward, 5′-TTCATCCGCTGCTGCATCCAAGG-3′ and reverse, 5′-GCAAAGCACTCCATGACGAA-3′; HOXA-AS3 (TV-2) forward, 5′-TGGCACCCAAATGTCATATAGC -3′ and reverse, 5′-ATTTACCGACTGCTTACACTGATG -3′; HOXA3, forward, 5′-CCAAGCTCACCCACCTGTGA-3′ and reverse, 5′-AAAGAAGGTCGGGTGGGCAA-3′; HOXA5, forward, 5′-ATGGCCCGGACTACCAGTTG -3′ and reverse, 5′-TGTAGCCGTAGCCGTACCTG -3′; HOXA6 forward, 5′-AGTCTCCCGGACAAGACGTA-3′ and reverse, 5′- GGCTGCGTGGAATTGATGAG-3′; NF110 forward, 5′-CTGCTAAGCCACATAACCC -3′ and reverse, 5′- GTTGCCTCCGTACCCATA-3′; GAPDH forward,5′-CAATGACCCCTTCATTGACC-3′ and reverse,5′- TGGAAGATGGTGATGGGATT-3′; ACTIN forward, 5′-CTCCATCCTGGCCTCGCTGT-3′ and reverse,5′- GCTGTCACCTTCACCGTTCC -3′; U1 snRNA forward, 5′-GGGAGATACCATGATCACGAAGGT -3′ and reverse,5′- CCACAAATTATGCAGTCGAGTTTCCC-3′. The qRT-PCR assays were conducted in an ABI 7500 apparatus (Applied Biosystems, Foster City, CA, USA). The amplification reaction was performed for 1 min at 95°C, 15 min at 95°C, followed by 40 cycles at 95°C for 15 s and 52°C for 15 s. The relative quantification of target genes expression was relative to the GAPDH levels. All qRT-PCR reactions were performed in duplicate.

**Isolation of cytoplasmic and nuclear RNA**
Cytoplasmic and nuclear RNA were isolated and purified using the PARIS^™^ Kit (Thermo Fisher Scientific, Scotts Valley, CA, USA) according to the manufacturer’s instructions. The nuclear and cytoplasmic RNAs were then converted to cDNA and analyzed by qRT-PCR.

**RNA-FISH**
Fluorescence-conjugated lncHOXA-AS3 probes were used for RNA-FISH, which was performed as previously described [^1^](#_ENREF_1). Hybridization was performed using DNA probe sets (Ribobio, Guangzhou, China) according to the manufacturer’s instructions, and control cells were observed using an NA1.4 inverted Leica DMI6000 microscope (Leica, Heidelberg, Germany). The images were recorded using a Hamamatsu ORCA-R2 camera (Hamamatsu Photonics, Hamamatsu, Japan) and recorded by LAS AF software (Leica).

**siRNA transfection of A549 cells**

The expression of HOXA-AS3, HOXA6, NF110 were silenced by transfecting A549 cells with small interfering RNA, which was designed and synthesized by GenePharma. A non-targeted control siRNA (siNC) was used as a negative control to determine and optimize the efficiency of transfection. The detailed siRNA sequences were listed as follows, the used si/HOXA-AS3-1 sequence was 5′-UCUAUUCUCGCAAGGGAAATT -3′, si/HOXA-AS3-2 sequence was 5′-GGUAGAUUCAUAGAAUAUAAC -3′, si/HOXA6 sequence was 5′-GGACAAGACGUACACCUCACC -3′, si/NF110-1 sequence was 5′-CCAGAUGGUUCUGGCAUUUTT -3′, si/NF110-2 sequence was 5′-CCUGUGUGAGAAAUCCAUUTT -3′ and the siNC sequence was 5′-UUCUCCGAACGUGUCACGUTT-3′. siRNA was transfected into A549 cells using the Lipofectamine 2000 siRNA transfection reagent following the manufacturer’s protocol. In brief, A549 cells were used at a confluence of 50%–70%;1.5 mg of siRNA and 7.5 ml of the Lipofectamine 2000 siRNA Transfection Reagent were separately diluted in 100 μL of serum-free Opti-MEM-1 medium for 5 min, mixed and incubated at room temperature for 20 min. The siRNA-transfection reagent mixture was added directly to the cells. Cells were quiescenced for 24 h and used as required.

**Bromodeoxyuridine (BrdU) incorporation**A549 cells were plated into 96-well plates at a density of 1 × 10^4^ cells/well, and then subjected to growth arrest for 24 h before siRNA transfection. BrdU was incorporated into proliferating cells by adding at 24 h prior to the end of the test reagent incubation. Following incorporation, the cells were fixed using a fixing solution at room temperature for 30 min. The following steps were done according to the manufacturer’s protocol. Finally, the plate was read using a spectrophotometer microplate reader at dual wavelengths of 450/550 nm.

**Western blot analysis**

Proteins were solubilized and extracted with lysis buffer (Tris 50 mM, pH 7.4, NaCl 150 mM, Triton X-100 1%, EDTA 1 mM, and PMSF 2 mM) and incubated for 30 min on ice. The lysates were sonicated and centrifuged at 16,099 g for 15 min and the insoluble fractions were discarded. Protein concentrations were determined by the Bradford assay using bovine serum albumin (BSA) as standard. Cell samples containing 20 μg of protein were subjected to electrophoresis on an SDS-polyacrylamide gel as previously described. After electrophoresis, proteins were transferred to nitrocellulose membranes. These membranes were blocked in blocking buffer (Tris 20 mM, pH 7.6, NaCl 150 mM and Tween 20 0.1%) containing 5% nonfat dry milk and incubated with PCNA (Boster, 1:400), cyclinA (Boster, 1:200), cyclinD (Boster, 1:200), cyclinE (Boster, 1:100), CDK1 (Boster, 1:100), CDK2 (Boster, 1:100), CDK4 (Boster, 1:100), HOXA3 (Boster, 1:100), HOXA5 (Boster, 1:100) HOXA6 (Boster, 1:100), P53 (Boster, 1:200), NF110 (Abcam, 1:100), β-actin (Santa Cruz Biotechnology, 1:2000) and secondary antibodies as described previously. The proteins were visualized with enhanced chemiluminescence reagents (SuperSignal, Pierce, Rockford, IL, USA).

**Immunofluorescence assay**
Immunohistochemistry was conducted using a technique described previously [^2^](#_ENREF_2).

The cells were incubated with an antibody against ki67 (Santa Cruz Biotechnology

Santa Cruz, CA, USA) (1:100) overnight at 4°C, incubated with secondary IgG (Santa Cruz Biotechnology) (1:1000) conjugated with FITC for 1 h in the dark at 37°C, and washed with PBS. The nuclei were stained with DAPI (4,6-diamidino-2-phenylindole). The slides were examined using NA1.4 inverted Leica DMI6000 microscope (Leica, Heidelberg, Germany), images were visualized by Hamamatsu ORCA-R2 camera (Hamamatsu, Japan) and recorded by LAS AF software (Leica, Heidelberg, Germany). The experiments were conducted in triplicate.

**The 3-(4,5-dimethylthiazol-2-yl)-2,5-diphenyltetrazolium bromide** (**MTT) assay**

The cell viability was determined using the MTT assay as previously described [^3^](#_ENREF_3). The reaction was terminated by adding dimethylsulfoxide to the medium, followed by incubation for 10 min at 37°C. The absorbance was read at 490 nm using a spectrophotometer, to determine the cell viability.

**Cell cycle analyses**The proportions of cells in the G0/G1, S, and G2/M phases were detected by flow cytometry. Briefly, the cells were harvested by trypsinization and then fixed with 70% ethanol at 4°C. The fixed cells were centrifuged at 300 × g for 5 min and resuspended in 500 μL staining buffer before detection. A total of 10 μL of RNaseA was added and mixed, then 25 μL of propidium iodide was added, and the suspension was incubated in a 37°C in a water bath for 30 min. Finally, the cells were filtered once through 400-mesh sieves and detected by flow cytometry.

**Scratch-wound assay**

The six-well plates on which fully confluent A549 cells were cultured were scratched with a fine sterile pipette tip to generate a cell-free gap with a width of 1 mm, and the damaged cells were removed using PBS. After that, the cells were treated with vehicle or the chemicals of interest in 5% FBS-DMEM. The cells in all study groups were photographed in the same area of the culture plate immediately after and 24 h after the wound.

**Transwell^®^ assay**

Cell migration was measured using a Matrigel^®^-coated modified Boyden chamber with a polycarbonate filter with a pore size of 8 μM. After 24 h of incubation, the nonmigrating cells in the upper chamber were removed. The cells on the underside of the membrane were incubated in 4% formaldehyde solution for 10 min, followed by incubation in 0.4% Crystal Violet in 10% ethanol for 5 min. The number of migrated cells was measured by counting the number of stained nuclei per high power field in a microscope (Olympus, Tokyo, Japan). Each sample was counted randomly at nine separate locations in the center of the membrane, and the A549 cell migration activity was reported as the number of cells migrating per field of view. The experiments were performed at least three times in quadruplicate.

**Plasmid constructions**

Full-length HOXA-AS3 and GAPDH were cloned into the eukaryotic expression vector pcDNA3.1 with C-terminal Flag tag and these plasmids were transfected into A549 cells separately with Lipofectamine 2000, according to the manufacturer’s instructions. After 48 hours, immunoblotting was used to detect the Flag-tagged protein.

**Lentiviral Vector Transfection**

The shRNA targeting HOXA-AS3 (sh/HOXA-AS3) or NC were provided by Genechen (Shanghai, China). For overexpression assay, HOXA6 (Lv-HOXA6) or vector (Lv-Ctrl) were provided by Genechen (Shanghai, China). Lentiviral particles were used to infect A549 cells according to the manufacturer's instructions. Transduction efficiency (MOI=100) was determined by GFP expression to be over 80% and knockdown efficiency was measured by qPCR to be about 70% down.

**Colony formation assay**

The transduced and control cells were placed in 6-well plates at a density of 500 cells/well and maintained in medium containing 10% FBS. The culture medium was replaced every 4 days during the growth of colonies. After 14 days, the cell colonies were washed with phosphate-buffered saline, fixed with 4% paraformaldehyde for 10 min, and stained with Crystal Violet for 8 min. Triplicate wells were measured for each treatment group.

**The tumor formation assay in nude mice**

Male BALB/c nude mice (6-weeks-old) were purchased from Vital River Laboratories (Beijing, China) and maintained in pathogen-free conditions. The mice were injected subcutaneously into both flanks with 1 × 10^8^ cells/mL of A549 cells, which were stably transfected with sh/HOXA-AS3 or the empty vector. Tumor growth was examined every 3 days, and tumor volumes were calculated using the equation, V = 0.5 × length × width^2^. At 28 days after injection, the mice were euthanized, and the subcutaneous growth of each tumor was examined by western blotting and hematoxylin and eosin (H&E) staining. Tumor sections were fixed with 4% paraformaldehyde overnight, dehydrated, cleared, and embedded in paraffin wax. Tumor sections with a thickness of 5 μM were prepared and stained with H&E stain. The protocol was approved by the Institutional Animal Care and Use Committee.

**mRNA stability assay**Cells were incubated for the indicated times following the addition of 5µg/mL actinomycin D (Sigma Chemical Co.) as previously described [^4^](#_ENREF_4). Total RNA was subsequently extracted and qRT-PCR was conducted to quantify the levels of HOXA3, HOXA5 and HOXA6 mRNA.

**RNase protection assay**As previously described [^5^](#_ENREF_5), each RNA sample was extracted from cells, incubated for 1 h at 37°C, and then treated with RNase A + T (Sigma-Aldrich, St. Louis, MO, USA) for 30 min at 37°C. RNA was extracted and analyzed using PCR.

**Microscale thermophoresis (MST)**For MST experiments, as previously described [^6^](#_ENREF_6), a Monolith NT.115 system (NanoTemper Technologies, Munich, Germany) was used. A 15% infrared laser with 20% light emitting diode power was applied for generating local temperature gradients and illumination, respectively. Laser on and off times were set at 60 seconds and 10 seconds, respectively. Cold fluorescence was averaged over a time period of 5 seconds before the temperature jump, and the warm fluorescence signal was averaged over 30 seconds, starting at 5 seconds after the temperature jump. Approximately 5 μL sample volumes were placed into standard treated capillaries (NanoTemper Technologies, South San Francisco, CA, USA) for measurements.

**RNA pull-down assay**

The RNA pull-down assay was performed using the Magnetic RNA-Protein Pull-Down Kit (Pierce, Waltham, MA, USA) according to the manufacturer's instructions. First, the full-length HOXA-S3 was synthesized using the RiboMAX^™^ Large Scale RNA Production System (Promega, Madison, WI, USA). After biotin labeling, the HOXA-AS3 was bound to the beads for protein binding. The cell protein lysate was added with RNA-bound beads for immunoprecipitation, and the beads were washed three times and boiled in SDS buffer, followed by detection of the retrieved protein using western blot analyses.

**RNA immunoprecipitation (RIP) Assay**

RIP was performed with the Magna RIP RNA-Binding Protein Immunoprecipitation Kit following the manufacturer’s instructions as described [^7^](#_ENREF_7)^,^ [^8^](#_ENREF_8). HOXA-AS3, SNRNP70 and IgG antibodies were used. Final analysis was performed using qRT-PCR and shown as fold enrichment of HOXA-AS3 and U1.

**ChIP assay**A549 cells or 16HBE cells were fixed in formaldehyde, then sonicated to prepare the chromatin fragments. The genomic DNA fragments were immunoprecipitated with antibodies against H3K9me3, H3K9ac, or normal rabbit IgG at 4°C for 3 h. After crosslinking reversal and DNA cleanup, the purified and precipitated DNA was analyzed by qRT-PCR.

**References**

1. Wang P. *et al.* The STAT3-binding long noncoding RNA lnc-DC controls human dendritic cell differentiation. *Science* 2014, **344**(6181)**:** 310-313.

2. Liu Y. *et al.* The key role of transforming growth factor-beta receptor I and 15-lipoxygenase in hypoxia-induced proliferation of pulmonary artery smooth muscle cells. *The international journal of biochemistry & cell biology* 2012, **44**(7)**:** 1184-1202.

3. Song S. *et al.* Biliverdin reductase/bilirubin mediates the anti-apoptotic effect of hypoxia in pulmonary arterial smooth muscle cells through ERK1/2 pathway. *Experimental cell research* 2013, **319**(13)**:** 1973-1987.

4. Kang S. *et al.* TGF-beta Suppresses COX-2 Expression by Tristetraprolin-Mediated RNA Destabilization in A549 Human Lung Cancer Cells. *Cancer research and treatment : official journal of Korean Cancer Association* 2015, **47**(1)**:** 101-109.

5. Sun J. *et al.* Long noncoding RNA FGFR3-AS1 promotes osteosarcoma growth through regulating its natural antisense transcript FGFR3. *Molecular biology reports* 2016, **43**(5)**:** 427-436.

6. Lippok S. *et al.* von Willebrand factor is dimerized by protein disulfide isomerase. *Blood* 2016, **127**(9)**:** 1183-1191.

7. Nie FQ. *et al.* Long noncoding RNA ANRIL promotes non-small cell lung cancer cell proliferation and inhibits apoptosis by silencing KLF2 and P21 expression. *Molecular cancer therapeutics* 2015, **14**(1)**:** 268-277.

8. Wu G. *et al.* LincRNA-p21 regulates neointima formation, vascular smooth muscle cell proliferation, apoptosis, and atherosclerosis by enhancing p53 activity. *Circulation* 2014, **130**(17)**:** 1452-1465.
