# Supplementary material for: Increased levels of the long noncoding RNA, HOXA-AS3, promote proliferation of A549 cells
Source: Cell Death Dis. 2018 Jun 13;9(6):707. doi: 10.1038/s41419-018-0725-4 (PMC5999602; doi:10.1038/s41419-018-0725-4)
Supplement: Supplementary file 2 — Supplmentary Data 2 [file 41419_2018_725_MOESM2_ESM.docx]

**Supplemental Figures**

**Figure S1**

**
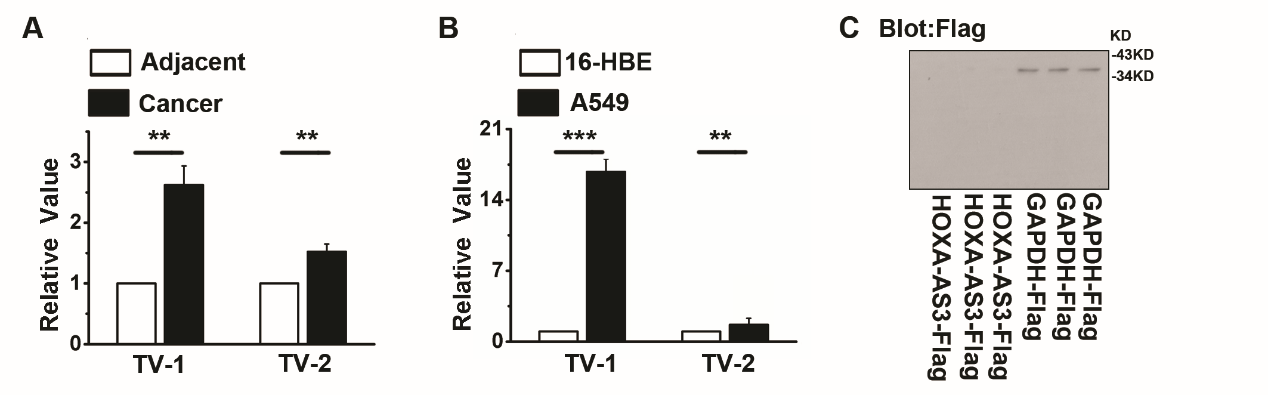
**

**Figure S1. HOXA-AS3 transcript variants showed differential up regulation of expression.** A, Expression of TV-1 and TV-2 quantified by the quantitative polymerase chain reaction (qRT-PCR) in LAD samples and their corresponding adjacent normal tissues. B, Expression of TV-1 and TV-2 quantified by qRT-PCR in human LAD cell line, A549, and in a normal bronchial epithelial cell line, 16HBE. C, HOXA-AS3 is a long non-coding RNA. *P < 0.05, ^**^P < 0.01, and ^***^P < 0.001. All values are expressed as the mean ± SEM.

**Figure S2**

**
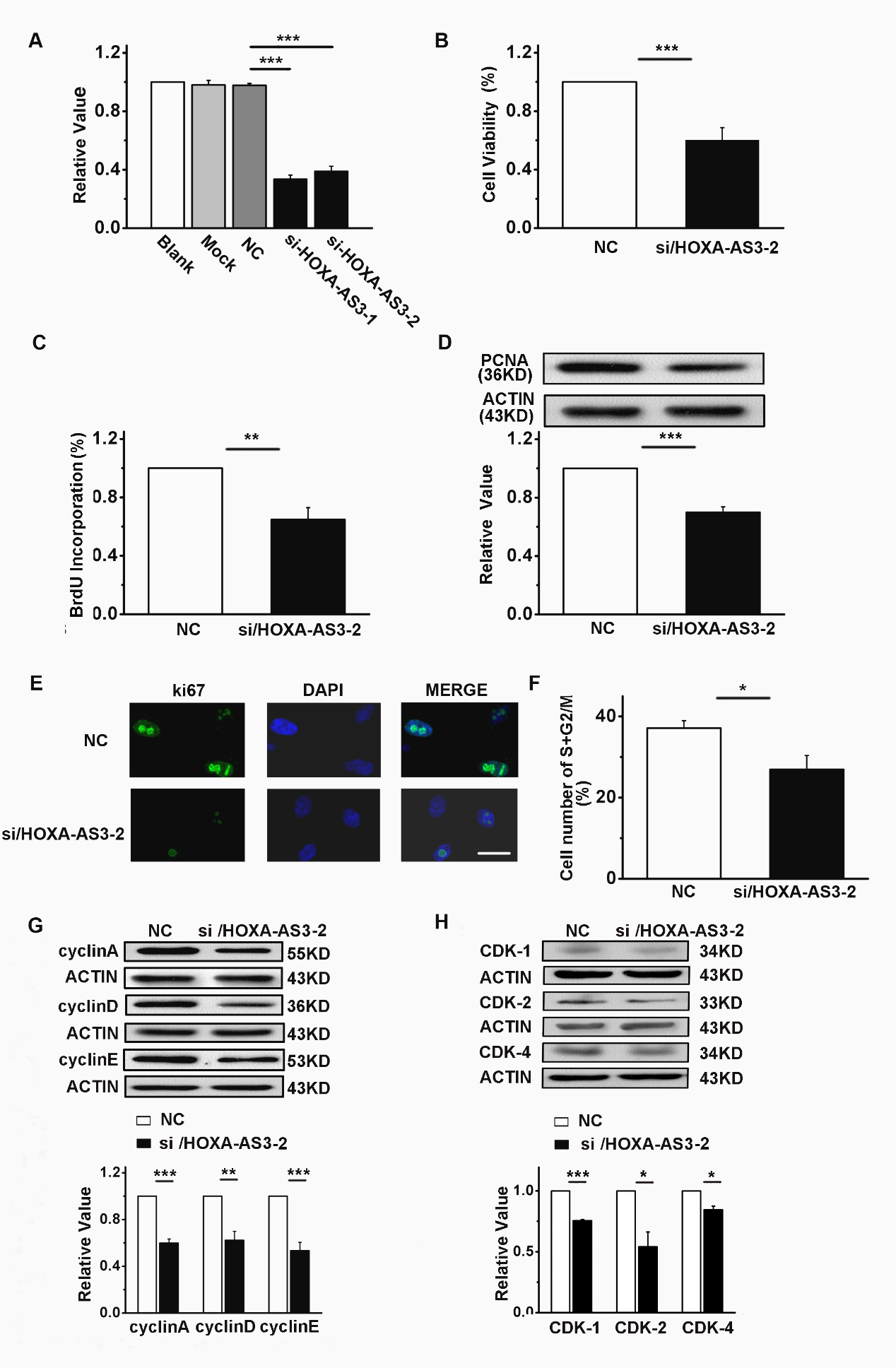
**

**Figure S2. HOXA-AS3 knockdown reduces cell proliferation and** **regulates the cell cycle.** A, The efficiency and specificity of siRNA directed against HOXA-AS3 using the qRT-PCR. B, The 3-(4,5-dimethylthiazol-2-yl)-2,5-diphenyltetrazolium bromide (MTT) assay was used to determine the cell viability in A549 cells. C, 5-bromodeoxyuridine incorporation showed the synthesis of DNA. D, Western blot analyses of the protein expression levels of proliferating cell nuclear antigen. E, Immunofluorescence for ki67 in A549 cells, after staining the nuclei with 4',6-diamidino-2-phenylindole (DAPI). Scale bar, 25 μm. F, Fluorescence-activated cell sorting (FACS) analyses to determine the percentage of cells in S and G2/M phases in A549 cells. G, Western blot analyses of the protein expression levels of cyclin A, cyclin D, and cyclin E. H, Western blot analyses of the protein expression levels of CDK1, 2, and 4. NC, negative control; siRNA/HOXA-AS3, small interfering RNA for HOXA-AS3. ^*^P < 0.05, ^**^P < 0.01, and ^***^P < 0.001. All values are expressed as the mean ± SEM.

**Figure S3**


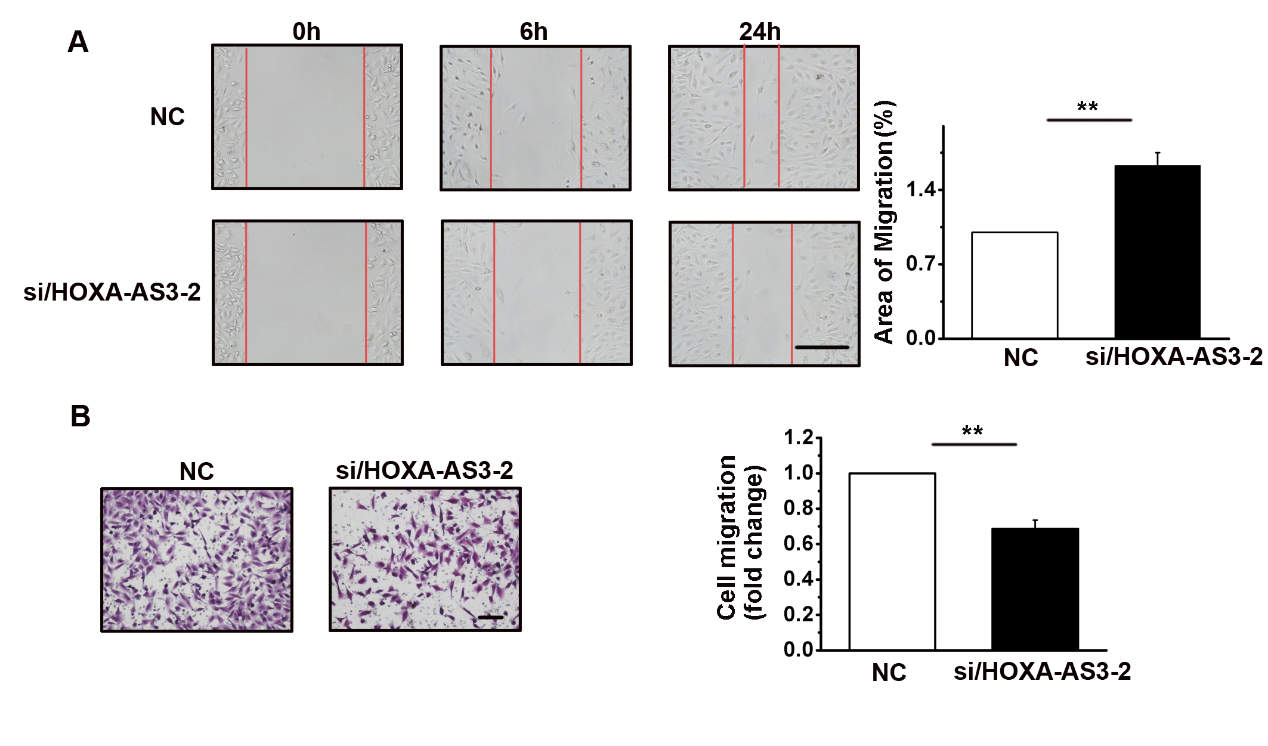


**Figure S3. HOXA-AS3 knockdown inhibits cell migration and invasion in A549 cells.** A, Left, A549 cells were subjected to a scratch-wound assay. Scale bar, 50 μm. Right, the histogram shows the cell migration ability of A549 cells. B, Left, A549 cells were subjected to Matrigel^®^ invasion chamber analyses. Scale bar, 50 μm. Right, the histogram shows the cell invasion ability of A549 cells. NC, negative control; siRNA/HOXA-AS3, small interfering RNA for HOXA-AS3; ^*^P < 0.05, ^**^P < 0.01, and ^***^P < 0.001. All values are expressed as the mean ± SEM.

**Figure S4**


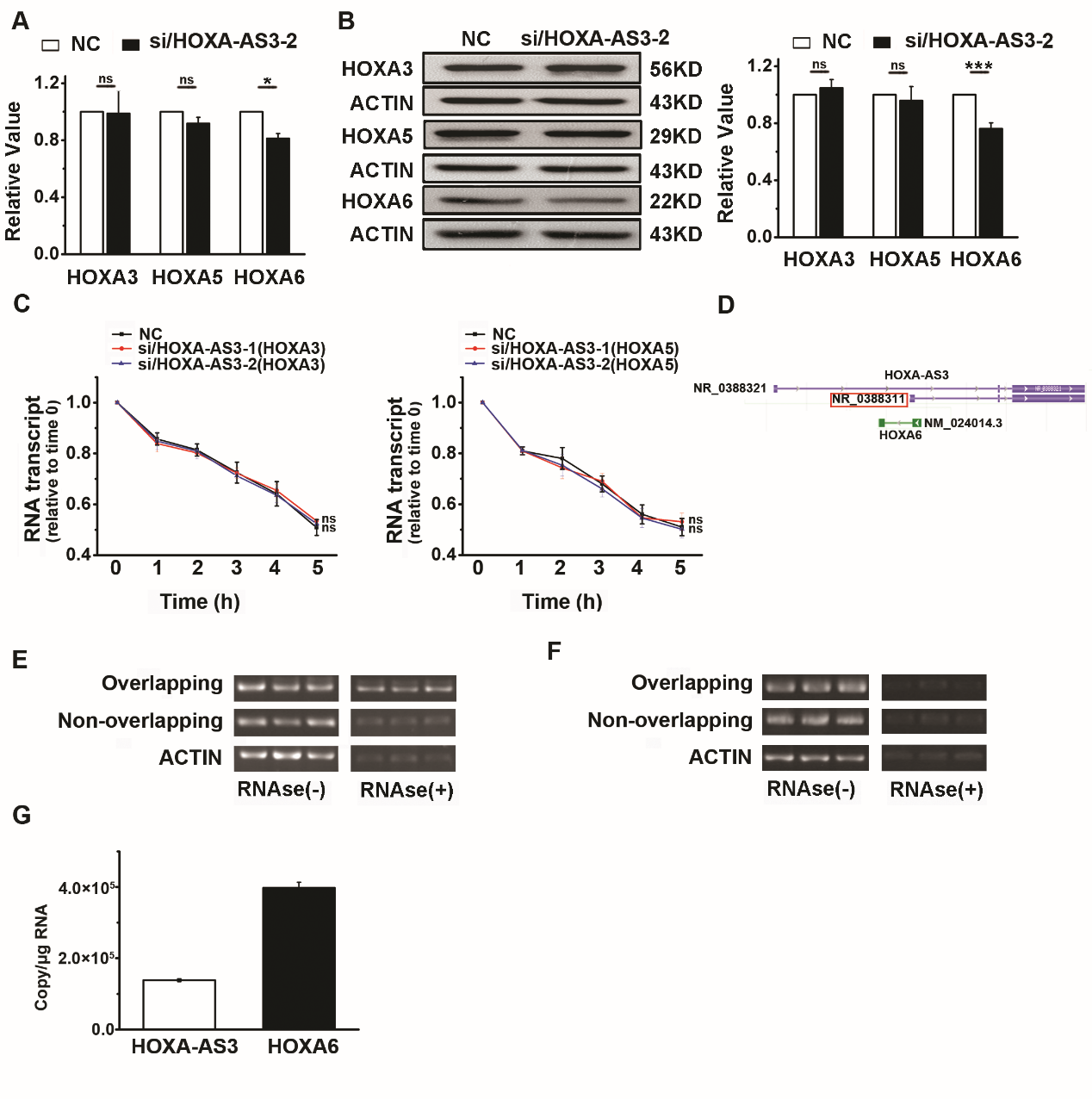


**Figure S4. HOXA-AS3 and HOXA6 mRNAs form a duplex RNA-RNA structure at their mutually overlapping regions.** A, Expression of HOXA3, HOXA5, and HOXA6 quantified by qRT-PCR after HOXA-AS3 knockdown in A549 cells. B, Western blot analyses of the protein expression levels of HOXA3, HOXA5, and HOXA6 after HOXA-AS3 knockdown in A549 cells. C, The stability of HOXA3 and HOXA5 mRNA over time was measured by qRT-PCR relative to time 0 after 5μg/ml actinomycin D treatment in A549 cells transiently expressing HOXA-AS3 siRNAs or control siRNA. D, Genomic sequences of HOXA-AS3 and HOXA6. as indicated on the UCSC site. E, RPA performed on RNA samples from A549 cells. RT-PCR results from two sets of primers and probes covering overlapping and nonoverlapping regions of HOXA6 mRNA. F, RPA performed on nuclear RNA samples from A549 cells. RT-PCR results from two sets of primers and probes covering overlapping and nonoverlapping regions of HOXA6 mRNA. G, The copy number of HOXA-AS3 and HOXA6 in A549 cells. ^*^P < 0.05, ^**^P < 0.01, and ^***^P < 0.001. All values are expressed as the mean ± SEM.

**Figure S5**

**
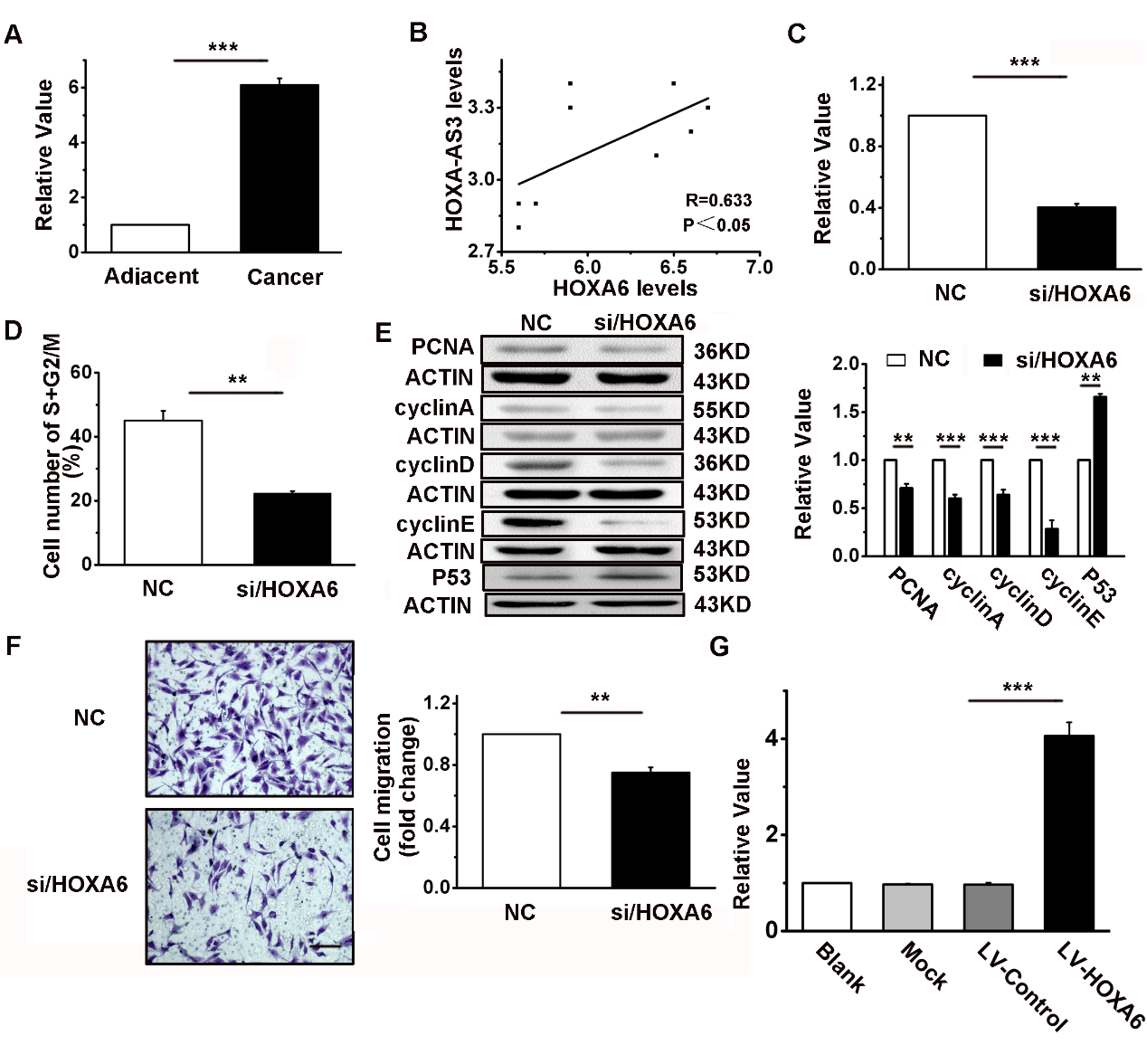
**

**Figure S5. HOXA6 knockdown inhibits cell proliferation, migration, and invasion in A549 cells.** A, Expression of HOXA6 quantified by qRT-PCR in lung adenocarcinoma (LAD) samples and their corresponding adjacent normal tissues. B, Correlation between HOXA-AS3 and HOXA6 mRNA expression in LAD tissues. C, Determination of the efficiency and specificity of siRNA directed against HOXA6 by qRT-PCR. D, Fluorescence-activated cell sorting (FACS) analyses to determine the percentage of cells in S and G2/M phases in A549 cells. E, Western blot analyses of the protein expression levels of proliferating cell nuclear antigen (PCNA), cyclin A, cyclin D, cyclin E, and P53 after HOXA6 knockdown in A549 cells. F, Left, A549 cells were subjected to Matrigel^®^ invasion chamber analyses. Scale bar, 50 μM. Right, the histogram shows the cell invasion ability of A549 cells. G, The expression of HOXA6 in stable A549 cells infected with lentiviruses expressing HOXA6. Adjacent, LAD corresponding to adjacent normal tissue; Cancer, LAD tissue; NC, negative control; siRNA/HOXA6, small interfering RNA for HOXA6. Blank, A549 cells were treated with DMEM; Mock, A549 cells were treated with polybrene; LV-Control, A549 cells were infected with empty lentiviruses; LV-HOXA6, A549 cells were infected with lentiviruses expressing HOXA6. *P < 0.05, **P < 0.01, and ***P < 0.001. All values are expressed as the mean ± SEM.

**Figure S6**

**
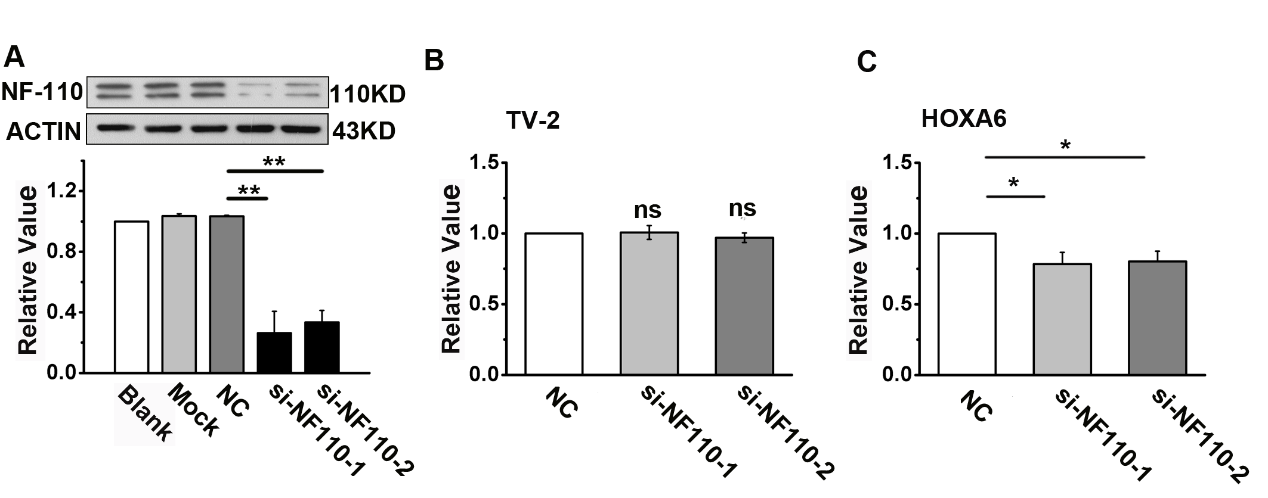
**

**Figure S6. NF110 knockdown decreases the expression of HOXA6.** A, Determination of the efficiency of siRNA directed against NF110 by qRT-PCR. B, Expression of TV-2 quantified by qRT-PCR after NF110 knockdown in A549 cells. C, Expression of HOXA6 quantified by qRT-PCR after NF110 knockdown in A549 cells. NC, negative control; siRNA/NF110, small interfering RNA for NF110. *P < 0.05, **P < 0.01, and ***P < 0.001. All values are expressed as the mean ± SEM.

**Figure S7**

**
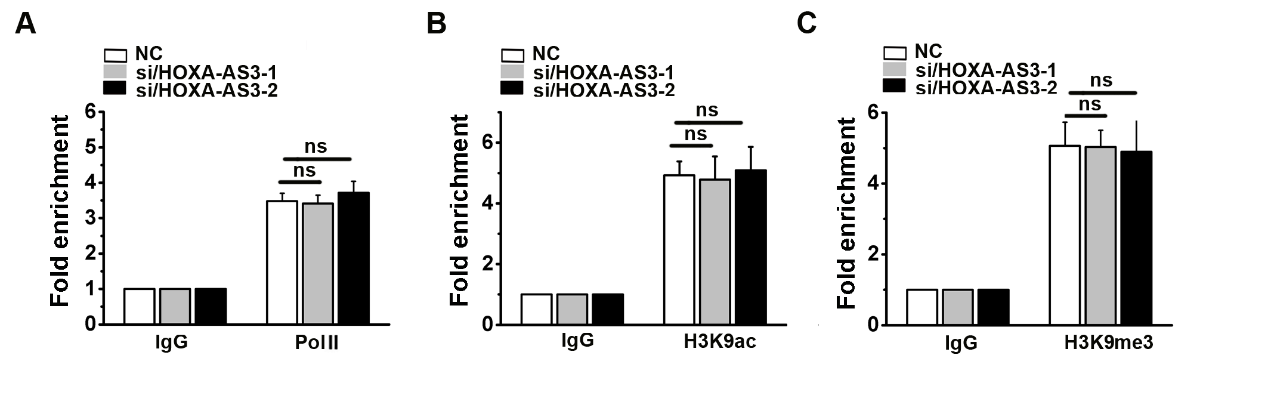
**

**Figure S7. HOXA-AS3 knockdown does not affect the histone modification of HOXA6 promoter region.** A-C, Binding of histone modifications to the HOXA6 promoter region was examined using a chromatin immunoprecipitation (ChIP) assay in A549 cells. ^*^P < 0.05, ^**^P < 0.01, and ^***^P < 0.001. All values are expressed as the mean ± SEM.
